# Intraspecific variation in pollination ecology due to altitudinal environmental heterogeneity

**DOI:** 10.1002/ece3.11553

**Published:** 2024-06-18

**Authors:** Gisela T. Rodríguez‐Sánchez, Roxibell C. Pelayo, Pascual J. Soriano, Tiffany M. Knight

**Affiliations:** ^1^ Instituto de Ciencias Ambientales y Ecológicas, Universidad de Los Andes Mérida Venezuela; ^2^ Laboratorio de Ecología Animal A, Departamento de Biología Universidad de Los Andes Mérida Venezuela; ^3^ Department of Microbial Population Biology Max Planck Institute for Evolutionary Biology Plön Germany; ^4^ German Centre for Integrative Biodiversity Research Leipzig Germany; ^5^ Department of Community Ecology Helmholtz Centre for Environmental Research—UFZ Halle (Saale) Germany; ^6^ Institute of Biology Martin Luther University Halle‐Wittenberg Halle (Saale) Germany

**Keywords:** Andes, environmental heterogeneity, flowers aggregation, *Oenothera epilobiifolia*, phenotypic variation, pollination ecotypes, self‐fertilisation

## Abstract

Plant‐pollinator interactions are constrained by floral traits and available pollinators, both of which can vary across environmental gradients, with consequences for the stability of the interaction. Here, we quantified how the pollination ecology of a high‐mountain hummingbird‐pollinated plant changes across a progressively more stressful environmental gradient of the Venezuelan Andes. We compared pollination ecology between two populations of this plant: Piedras Blancas (PB) and Gavidia (GV), 4450 and 3600 m asl, respectively. We hypothesised that self‐compatibility might be higher at the higher altitude site, however we found that flowers showed similar capacities for self‐compatibility in both localities. Seed production by flowers exposed to natural pollinators was significantly higher in the lower locality, where we also found higher nectar quality, larger flowers and increased frequencies of pollinator visitations. Interestingly, the population energy offered in the nectar was the same for both localities due to the higher density and floral aggregation found in the higher altitude population. Our study demonstrates how two plant populations in different environmental conditions have different pollination ecology strategies. Pollinator visitations or their absence result in trait associations in one population that are independent in the other. These population differences are not explained by differences in pollinator assembly, but by environmental heterogeneity.

## Graphical abstract

Plant populations in different eco‐regions differed in pollination ecology strategies. These population differences are not explained by differences in pollinator assembly, but by environmental heterogeneity.

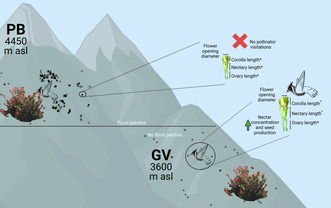

## 1 INTRODUCTION

A fundamental aim of ecological research is to understand how populations, communities and species interactions change along environmental gradients (Keddy, 1992; McGill et al., 2006). An important mechanism that can explain changes across environmental gradients is environmental filtering, in which only a subset of functional traits can persist in harsher environments (Junker & Larue‐Kontić, 2018; Korner, 2007). For example, plant traits are known to shift along altitudinal gradients, driven by the harsh filters of cold temperatures and other abiotic conditions that occur at high altitudes. To date, most of the research has focused on vegetative traits (Ladouceur et al., 2019; Rosbakh et al., 2022). Floral traits have received less attention, even though they have critical relevance for the pollination and reproductive success of plants (E‐Vojtkó et al., 2020, 2022). Floral traits within a plant species might vary across altitudinal gradients in a way that reflects local acclimatisation to abiotic conditions or change in the abundance and composition of pollinator communities (Ahmad et al., 2022; Heinrich & Raven, 1972; Petanidou et al., 1996). For example, the bee to fly transition that occurs with increases in altitude will lead to shifts towards different flower colours that are preferred by the local pollinator taxa (Bergamo et al., 2018; Renoult et al., 2015).

The onset of flowering is expected to be advanced at lower altitudes due to higher temperatures (Menzel et al., 2006). Flower longevity, however, has shown two trends, one the one hand it can be longer at lower altitudes due to warmer temperatures, but on the other hand, it can also be longer at higher altitudes as a mechanism to compensate for lower pollination success (Trunschke et al., 2017). Flower size has been shown to increase or decrease with altitude. Increases are often explained as a strategy to increase their attractiveness as a resource for the limited abundance of pollinators whereas decreases in flower size are often attributed to strategies for attracting smaller species of pollinators (Kudo, 2016) or to plant species relying more on selfing at higher altitudes (Sobrevila, 1989). Much less is known about changes in nectar across altitudes (Pyke & Waser, 1981). However, nectar quantity and quality are known to be sensitive to abiotic and biotic factors, such as temperature, water availability, light, nutrients, pollinators and microorganisms (Parachnowitsch et al., 2019). Nectar availability would be particularly important for mountainous bird species, which require high nectar volume (Stiles, 1978).

Given that there is a relationship between a set of floral traits and pollinator visitation (Faegri & van der Pijil, 1979), changes in floral traits are expected to have consequences for plant reproductive success. Pollinators often occur in lower abundances at high altitudes and this could either lead to pollen limitation, in which reproductive success of plants is limited by pollen receipt (Knight et al., 2005). Plants at high altitudes have been shown to have increased selfing for reproductive assurance (Körner & Paulsen, 2009). Floral aggregation is also known to influence pollinators foraging patterns and nectar availability (Ortiz‐Pulido & Vargas‐Licona, 2008). This resource‐availability‐dependent foraging makes the situation difficult to characterise as a cause or consequence of what is called bonanza‐blank patterns among patches, which means that not all flowers within a patch have the same resource volume (Zimmerman, 1981). If, for example, the offered nectar quality is low and the nutritional requirements of the pollinator are high, then its efficiency or visitation rate may decrease as a consequence of low‐energy reward for pollinators (McCallum et al., 2013).

A striking example of environmental gradient affecting pollination ecology is that of the emblematic plants ‘Frailejones’ (*Espeletiinae*, *Compositae*) in the Neotropical High Mountains. In these ecosystems some species are pollinated by wind and others by insects and hummingbirds (Berry & Calvo, 1989; Pelayo et al., 2019; Sánchez‐Guillén et al., 2015). In some species, mating systems change towards autogamy at the highest elevation (Berry, 1986), where the availability of pollinators is low (Sobrevila, 1989). Thus, we were interested in studying the pollination ecology of an ornithophilous plant at two altitudes. Focusing on a plant species with wide altitudinal distribution in combination with pollinators exclusion experiments is a useful strategy for testing pollinators effects on plant reproductive success via comparing the plant‐pollinator relationship and its effect on seed production at different altitudes.

We used the natural altitudinal gradient of the Venezuelan Andes and focus on *Oenothera epilobiifolia* (Onagraceae), a high‐mountain hummingbird‐pollinated plant species. This species is known to have corollas that turn from green to red after pollination (Wagner & Hoch, 2005). However, it is unknown whether flowers contain nectar at all stages and whether the different stages attract different pollinator assemblages (e.g., red should be more attractive to hummingbirds; Faegri & van der Pijil, 1979). We test whether the populations differ in their floral traits, their mating systems, their floral aggregation patterns and the frequency and composition of floral visitors. We assess whether there is a relationship between signal and reward in the two populations by correlating floral traits with nectar volume and concentration.

## 2 MATERIALS AND METHODS

### 2.1 Study system

**FIGURE 1:**
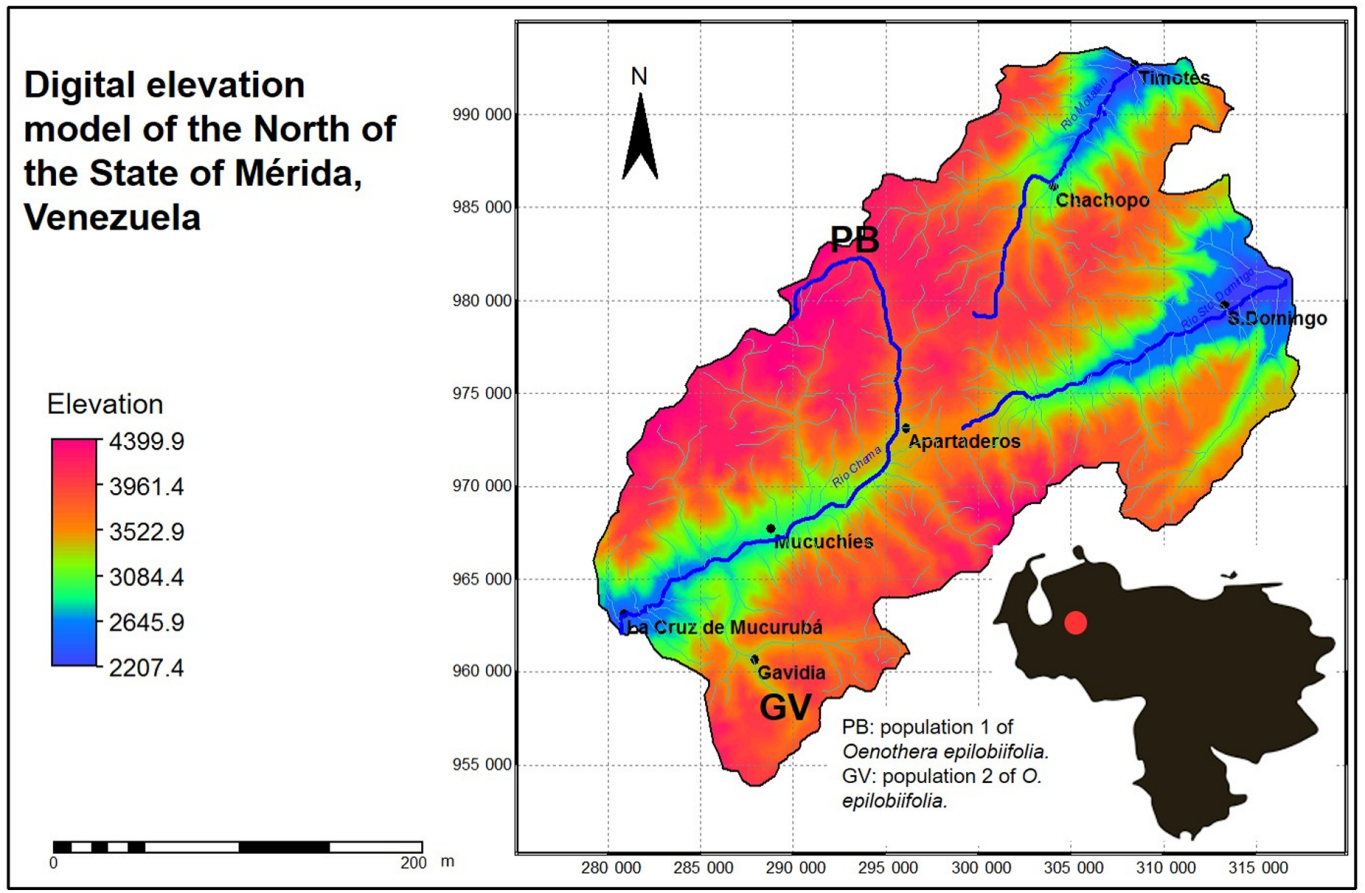
Location of *Oenothera epilobiifolia* populations studied in a digital elevation model from the north of Mérida state, Venezuela. Edited from Pelayo and Soriano (2010).

*Oenothera epilobiifolia* (Onagraceae) is found in Venezuela between 2000 and 4450 m asl (some bibliography report a maximum altitudinal range of 4100 m asl. However, we have found it at 4450 m asl). It is ornithophilous and has been shown to support the Venezuelan *Páramo* nectar feeders during June and July, just before the *Páramos* floral bloom (Pelayo et al., 2019). This is a perennial hermaphrodite herb with three distinguishable corolla floral types: green, red and orange (Briceño & Morillo, 2002; Vareschi, 1970). Berry (1986) found it to be auto‐compatible in some regions near 4100 m asl. known as the *Superpáramo*. We chose two populations located at different altitudes in Mérida State, Venezuela. The first population, *Piedras Blancas*, occurs at 4450 m asl (hereafter PB; UTM coordinates: 983,372, −292,355, Datum WGS 1984, zone 19) in *La Culata* mountain range and has daily cycles of freezing and thawing of the most superficial centimetres of the soil. The mean annual temperature ranges between 2.5 and (−2.0)°C and precipitation between 800 and 1200 mm per year. The second population, *Gavidia* (hereafter GV), is found in *Sierra Nevada* mountain range, it occurs at 3600 m asl (UTM coordinates: 958,236–289,875, Datum WGS 1984, zone 19) and has a mean annual temperature below 9°C, night frost and precipitation between 800 and 1800 mm per year (Ataroff & Sarmiento, 2004). The populations studied were within the La Culata and Sierra Nevada National Parks respectively (Figure 1).

### 2.2 Flower traits and pollinators assembly

The sampling was carried out between August and October 2018. To understand the dynamics of the three corolla types we followed the flower opening by tagging flowers of the different types to register the flowering time duration and corolla changes. At the same time, to determine floral traits, we selected a different set of flowers with a maximum of two flowers per plant per type for which we emptied nectar and bagged the flower, in total we sampled 60 flowers in PB and 111 flowers in GV. After 24 h, we made the following measurements on the green flowers only: the flower‐opening diameter, corolla, ovary and nectary length with an analogue vernier (Mauser), nectar volume with microcapillaries (0.5 and 1 μL; Drummond Scientific) and sucrose nectar concentration with an analogue refractometer (0–50°Brix, Eclipse, Bellingham & Stanley). We performed ln(*x* + 1) data transformation and compared each variable between both populations with Welch two‐sample *t*‐tests. To estimate signal and reward relationships, we also made Pearson correlations between morphological floral traits and nectar traits. All statistical tests were made in R 3.5.1 with dplyr package (R Core Team, 2022; Wickham et al., 2023); ggplot2, ggpattern and cowplot packages were used for plotting (Mike et al., 2022; Min, 2022; Wickham et al., 2018; Wilke, 2020). BioRender and ImageJ were also used for liustration and video editions, respectively (Schindelin et al., 2012).

To determine pollinator assemblies in each population, we used GoPro3 cameras and direct field observation to up to 37 and 55 h‐observation effort in PB and GV, respectively. Observations were made in open fields where several plants and pollinators were spotted; see below for more information on flower density and aggregation. We considered the visitation rate of each visiting animal as visits/10 min. It was our intention to compare visitation rate of plants in the two populations, however, one population received no pollinator visits (see Section 3).

Since we consider spatial floral pattern a fundamental component in plant‐pollinator interaction we designed the following set‐up with a different set of flowers to quantitatively compare both populations: In each locality, we established 6 5 × 5 m plots; each one was subdivided into 100 0.5 × 0.5 m mini‐plots. Using photographs and a reference system of each mini‐plot, we mapped the 2D spatial position of each flower within each plot. Then, to quantify and compare floral aggregation patterns we calculated the pair correlation function *g*(*r*), which determines the number of particles (for our purposes flowers) in concentric rings, from the centre of the ring to a distance *r* and compares it with the probability of that number of particles at the same distance *r* from the centre of the ring obtained by permutation of the previous data through a null randomisation model, we used the complete spatial randomness (CSR) model to simulate our null model (Crocker & Weeks, 2005). To gain statistical power, we used the WM estimator to combine the six replicate plots into a single analysis per population; this was done in the Programita Software (Wiegand & Moloney, 2004, 2014). Using the same flower maps, we also compared the flower relative density per flower stage between populations with Welch two‐sample *t* tests.

Then, using the statistically inferred nectar volume (PB *n = 18*, GV *n =* 48) and concentration (PB *n =* 17, GV *n* = 47) per flower in each population, the floral density (*n* = 6, 5 × 5 m plots per population) and the known energetic content of 1 M of sucrose solution, we calculated the nectar energy content of the whole population, previously correcting the volume of solution with *d =* (0.0037921 × *C*) + (0.0000178 × *C* ^2^) + 0.9988603, where *C* is the Brix degree from the refractometer (Hicks et al., 2016).

### 2.3 Reproductive system

To determine the mating system, we designed a two‐treatment experiment with a different set of flowers: (1) pollinator‐isolated flowers which were bagged before anthesis with tulle bags with pores less than 0.1 mm; and (2) pollinator‐exposed flowers which were bagged after the green phase, these flowers may or may not had been visited by pollinators. After 45 days we calculated and compared the seeds/initial ovules ratio for each flower between treatments and populations with a Wilcoxon rank sum exact test.

## 3 RESULTS

### 3.1 Flower types are not more than flower stages with different pollinator‐attractive traits

We learned that *O. epilobiifolia* does not have flowers of different corolla colours, but different flower stages. Our tagging experiment showed that flower colour changes throughout three phases each one characterised by a corolla colour and nectar availability. The flowering process begins with a green corolla for anthesis, then there is a phase with an orange‐yellow mosaic corolla (hereafter *changing*) and finally a red corolla. During this late stage, senescence occurs and fruit maturation begins. We hereafter refer to floral types as *stages*.

**FIGURE 2:**
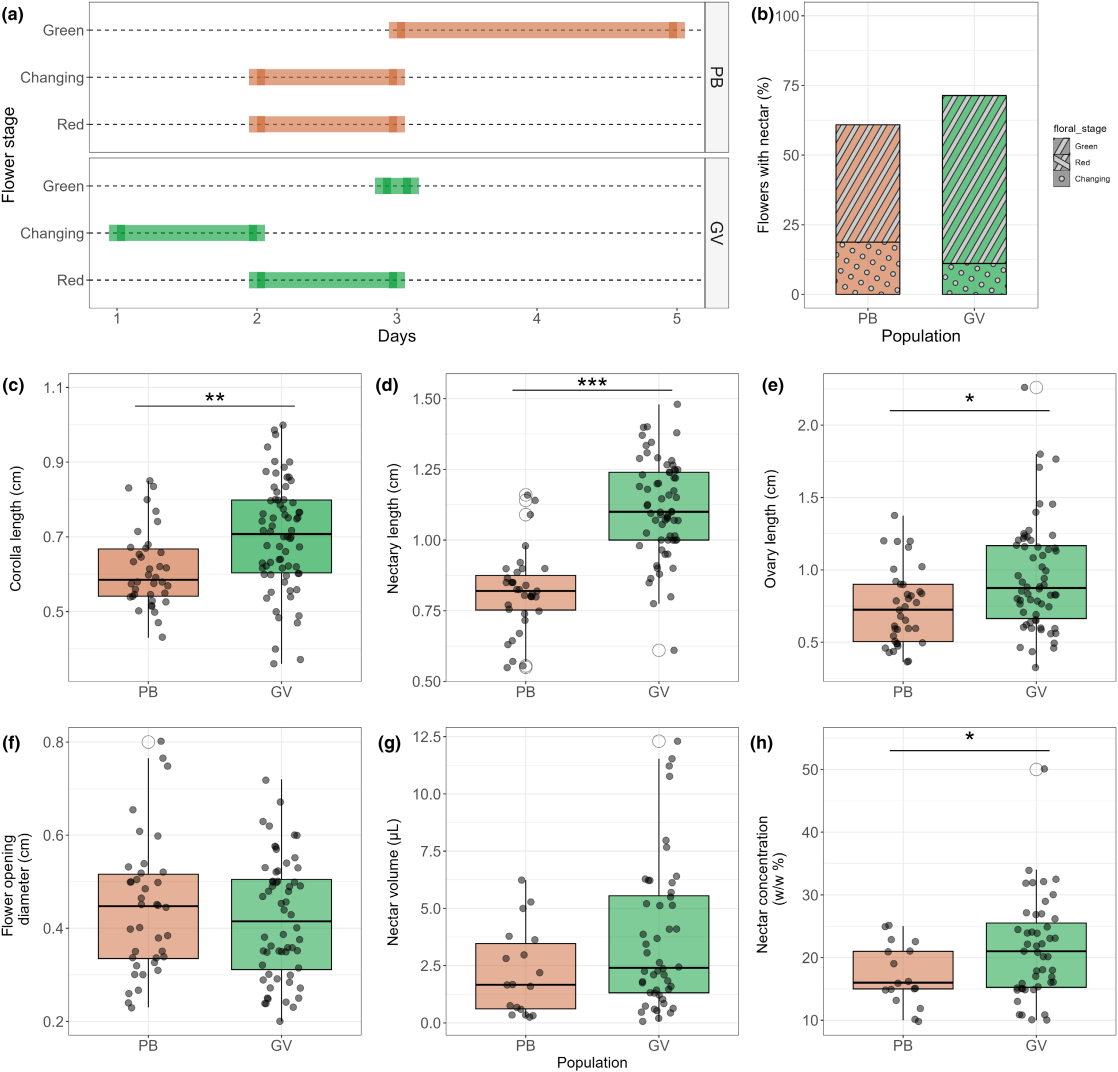
Pollination‐related flower traits for *Oenothera epilobiifolia* in the PB and GV populations. (a) Duration of each of the three flower stages. Anthesis initiates with the green stage and ends with the red stage when fruit maturation begins. Nectar is available only in the green stage. (b) Percentage of flowers with nectar available by stage in each population. (c–f) Morphological flower traits. (g, h) Nectar‐related traits. Welch two‐sample *t* test: *p‐value < .05, **p‐value < .001, ***p‐value < .00001.

Although flowers in both populations went through the three stages, the duration of the green stage was longer for PB (Figure 2a). Nectar availability also differed across stages and nectar was mainly present in the green stage. However, not all green flowers had nectar. In PB and GV, 42% and 60% of the green flowers presented nectar, respectively (Table 1 and Figure 2b).

**TABLE 1:** Sample sizes and absolute frequencies of flowers with and without nectar for each population.

Flower stage	PB		GV	
	Sampled flowers	Flowers with nectar	Sampled flowers	Flowers with nectar
Green	38	16	78	47
Changing	16	3	18	2
Red	6	0	15	0
Total	60	19	111	49

### 3.2 Flowers in the lower locality are larger and more attractive to pollinators

Pollination related traits such as floral size and sucrose concentration differ between populations. Corolla length, PB_*n* = 38_ (0.614 ± 0.104) cm, GV_*n* = 70_ (0.701 ± 0.144) cm; nectary length, PB_*n* = 35_ (0.818 ± 0.142) cm, GV_*n* = 65_ (1.114 ± 0.170) cm; and ovary length, PB_*n* = 37_ (0.741 ± 0.263) cm, GV_*n* = 62_ (0.915 ± 0.371) cm were significantly larger in the GV population. Interestingly, the flower‐opening diameter does not differ between populations PB_*n* = 38_ (0.444 ± 0.144) cm, GV_*n* = 60_ (0.420 ± 0.128) cm. Nectar volume was not different between the two populations either PB_*n* = 18_ (2.23 ± 1.90) μL, GV_*n* = 48_ (3.69 ± 3.19) μL. However, flowers in GV had nectar of higher sucrose concentration PB_*n* = 17_ (17.3 ± 4.9) w/w%, GV_*n* = 47_ (21.3 ± 7.8) w/w% (Figure 1c–h). For all cases: (mean ± standard deviation).

**FIGURE 3:**
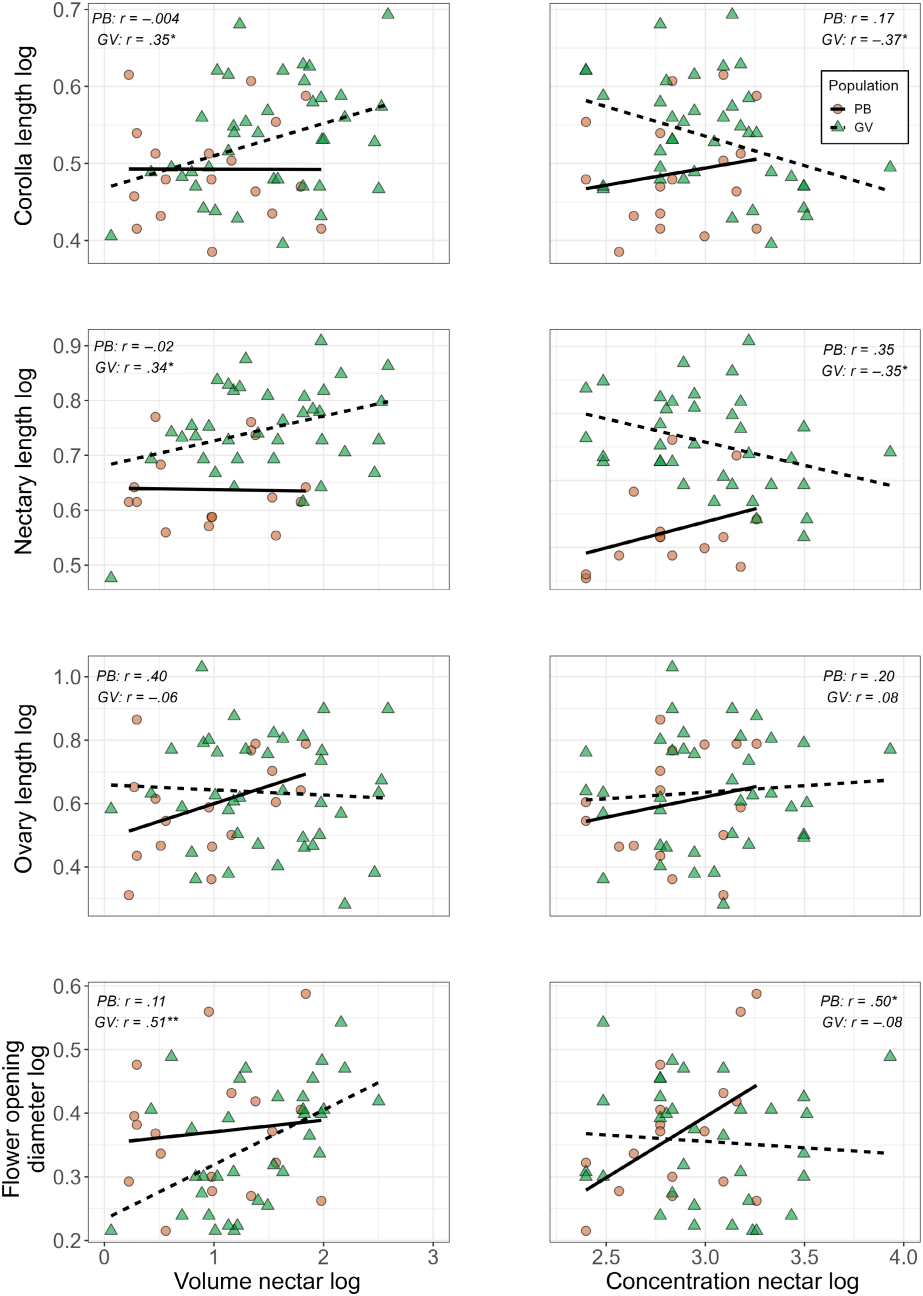
Association between morphological flower traits and nectar traits in the PB and GV populations. Traits were mainly positively associated with the volume of nectar only in the GV population, even though the mean volume of nectar itself was not significantly different between the two populations. Associations with nectar concentration showed different tendencies. Pearson's product–moment correlation*: *p*‐value < .05, **: *p*‐value < .001.

To investigate the relationship between signal and reward, we performed correlation tests between morphological floral traits and nectar traits. Impressively, although nectar volume did not show a significant difference between the two populations, only in GV there was a positive relationship between nectar volume and three floral traits: corolla length, nectary length and flower‐opening diameter. On the contrary, the nectar concentration is correlated only with two morphological flower traits and with different tendencies between traits and populations (Figure 3).

### 3.3 Pollinators visit flowers only in the lower locality

Although pollinators were present and actively visiting co‐flowering plant species in both populations (Videos 1, 2, 3, 4, 5, 6, 7), pollinators were only registered to visit our focal species in GV (0.46 visits/10 min, Videos 1 and 2). The visiting species included two hummingbirds (*Oxypogon lindenii* and *Metallura tyrianthina* with a visitation rate of 0.29 visits/10 min and 0.15 visits/10 min, respectively) and a Pieridae butterfly with a very low visitation rate (0.02 visits/10 min), which we consider a secondary visitor. Interestingly, we also registered an illegitimate visitation by *Diglossa gloriosa*, a common robber of nectar in these systems (Video 3). All visitation events took place only during the green flower stage.

**VIDEO 1:** Legitimate visit by *Metallura thrianthina* to *O. epilobiifolia* in the GV population.

**VIDEO 2:** Legitimate visit by *Oxypogon lindenii* to *O. epilobiifolia* in the GV population.

**VIDEO 3:** Illegitimate visit by *Diglossa gloriosa* to *O. epilobiifolia* in the GV population.

**VIDEO 4:** Multiple visits by *Colibri coruscans* to *Castilleja* sp.in the PB population.

**VIDEO 5:** Multiple visits by an unidentified hummingbird to *Castilleja* sp.in the PB population.

**VIDEO 6:** Oxypogon lindenii flying above *O. epilobiifolia* in the PB population.

**VIDEO 7:** Multiple visits by *Oxypogon lindenii* to *Castilleja* sp.in the PB population.

### 3.4 Flower density and aggregation are higher in the higher locality

Flower density was significantly variable among stages and populations, the green stage presented the higher density and density was always higher in the PB population (mean ± standard) flowers/m^2^, *n* = 6 (5 × 5 m) plots each population: Green stage: PB (5.83 ± 1.94), GV (1.97 ± 0.63). Changing stage: PB (0.97 ± 0.50), GV (0.27 ± 0.09). Red stage: PB (1.83 ± 0.59), GV (0.86 ± 0.44). Also, green flowers showed higher density compared to other stages (Figure 5a,b). The difference in floral aggregation including different flower stages was already visible in the plot maps (Figure 5a and Figures S1 and S2). The aggregation analyses confirmed that in PB, flowers were not only more aggregated than in GV, but the scale at which flower aggregation is maintained was also higher, this is the size of the flower patch at which aggregation of flowers is not explained by chance (Figure 5c).

### 3.5 Although nectar concentration differs, the population nectar offer is equal in both populations

Regarding the sucrose concentration in nectar, as expected, the energy content in nectar per flower in GV was higher than in PB, 3.32 cal and 1.50 cal, respectively. However, when we considered the nectar offered by the entire population in each locality, the energy content was very similar (PB = 4.10 cal/m^2^ and GV = 4.06 cal/m^2^). This occurred because in PB flower density was much greater. Thus, this situation seems to indicate that this plant population is offering the same energy content, but in different energy packages (Faegri & van der Pijil, 1979).

### 3.6 Pollinator visitations increase seed production

**FIGURE 4:**
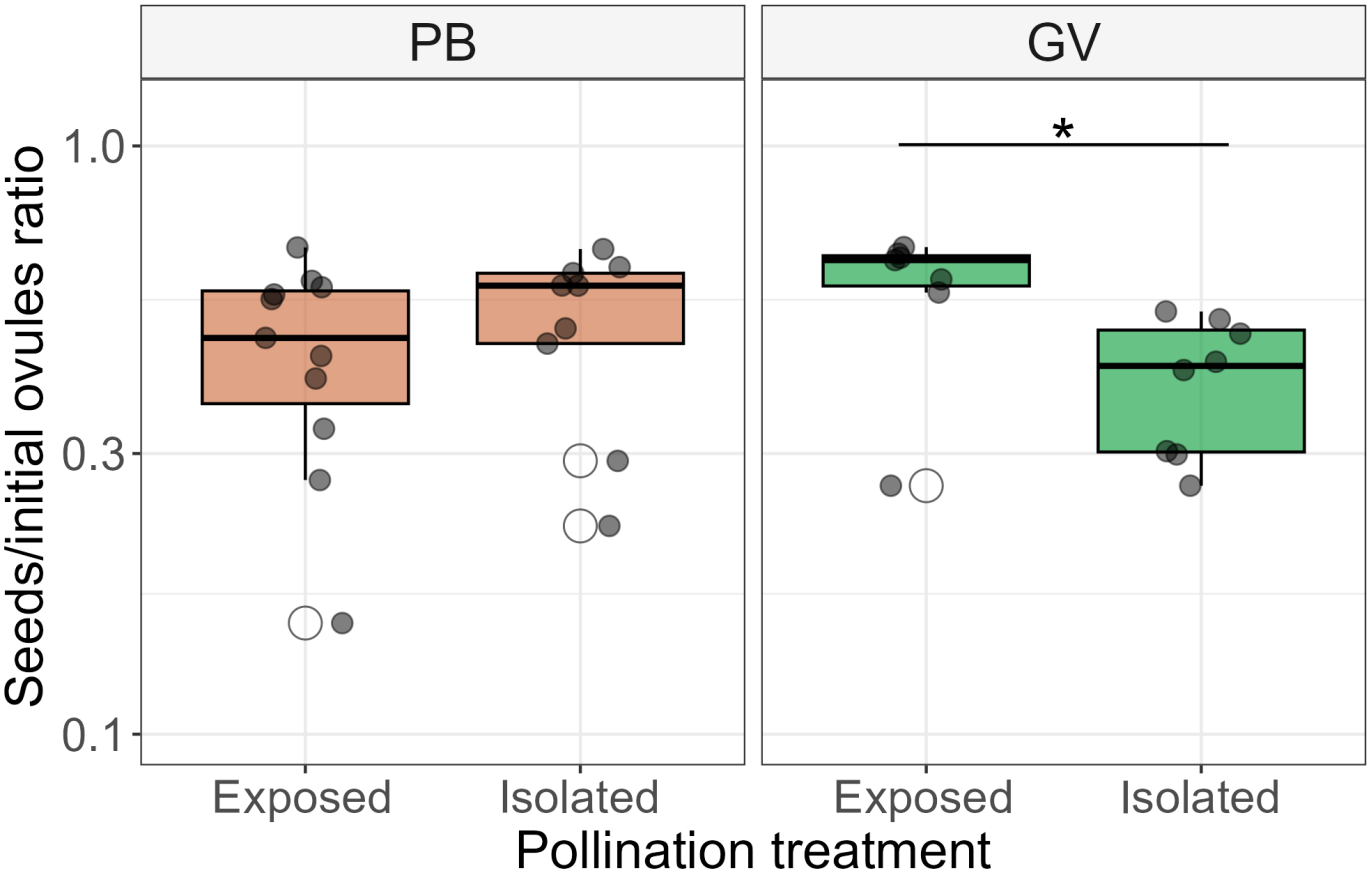
Seeds per ovules for different pollination treatments. Flowers exposed to pollinators had a significantly higher seed production compared to the isolated treatment only in the GV population. *: Wilcoxon rank sum exact test. *p*‐value < .05.

We found that flowers are self‐compatible with 50% seed/ovule efficiency in both populations in the bagged (isolated) treatment, (mean ± standard deviation) seed/ovule: PB_*n* = 11_ pollinators‐isolated (0.55 ± 0.34), GV_*n* = 9_ pollinators‐isolated (0.45 ± 0.21). However, only in GV, flowers exposed to possible pollinator visitations (pollinators‐exposed treatment) produced higher seed/ovule compared to pollinator‐isolated flowers. PB_*n* = 11_ pollinators‐exposed (0.59 ± 0.24), GV_*n* = 7_ pollinators‐exposed (0.79 ± 0.22), (Figure 4).

**FIGURE 5:**
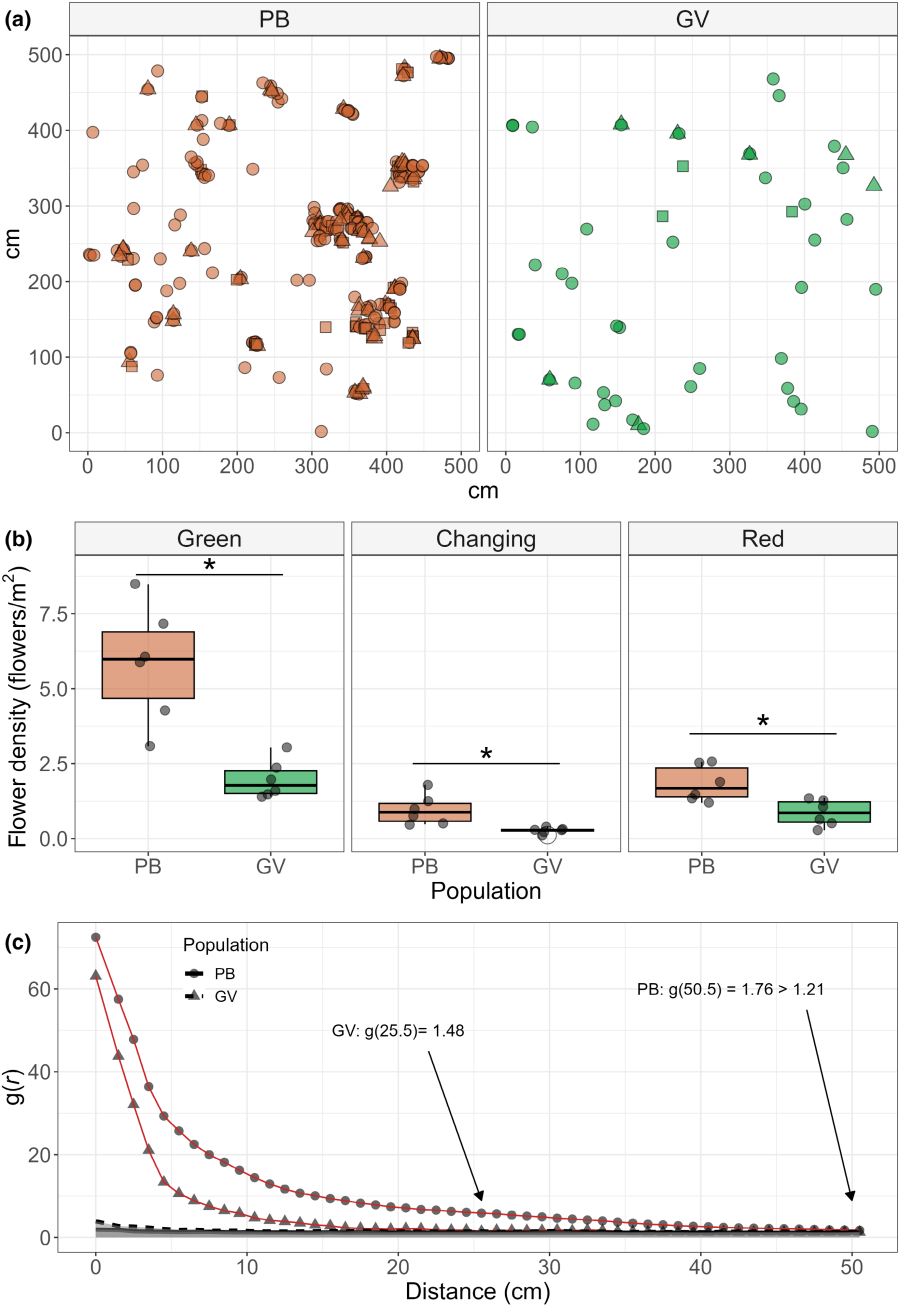
Flower density and aggregation in the PB and GV populations. a: Maps of 5 × 5 meter plots. Points represent flowers and shape flower stage: (a) circle, triangle and square for green, changing and red stages, respectively. (b) Flower density across the six map plots. PB population showed higher flower density for all flower stages. (c) Aggregation analysis, pair correlation function g(*r*) for all flower stages regardless of the corolla stage. Black arrows indicate the distance (*r*, diameter of the patch) at which the aggregation begins to be explained by chance. The scale at which aggregation is not explained by chance is higher in the PB population. In all cases, the confidence interval of the random null model was 95%. Welch two‐sample *t* test: *: *p*‐value < .05.

**FIGURE 6:**
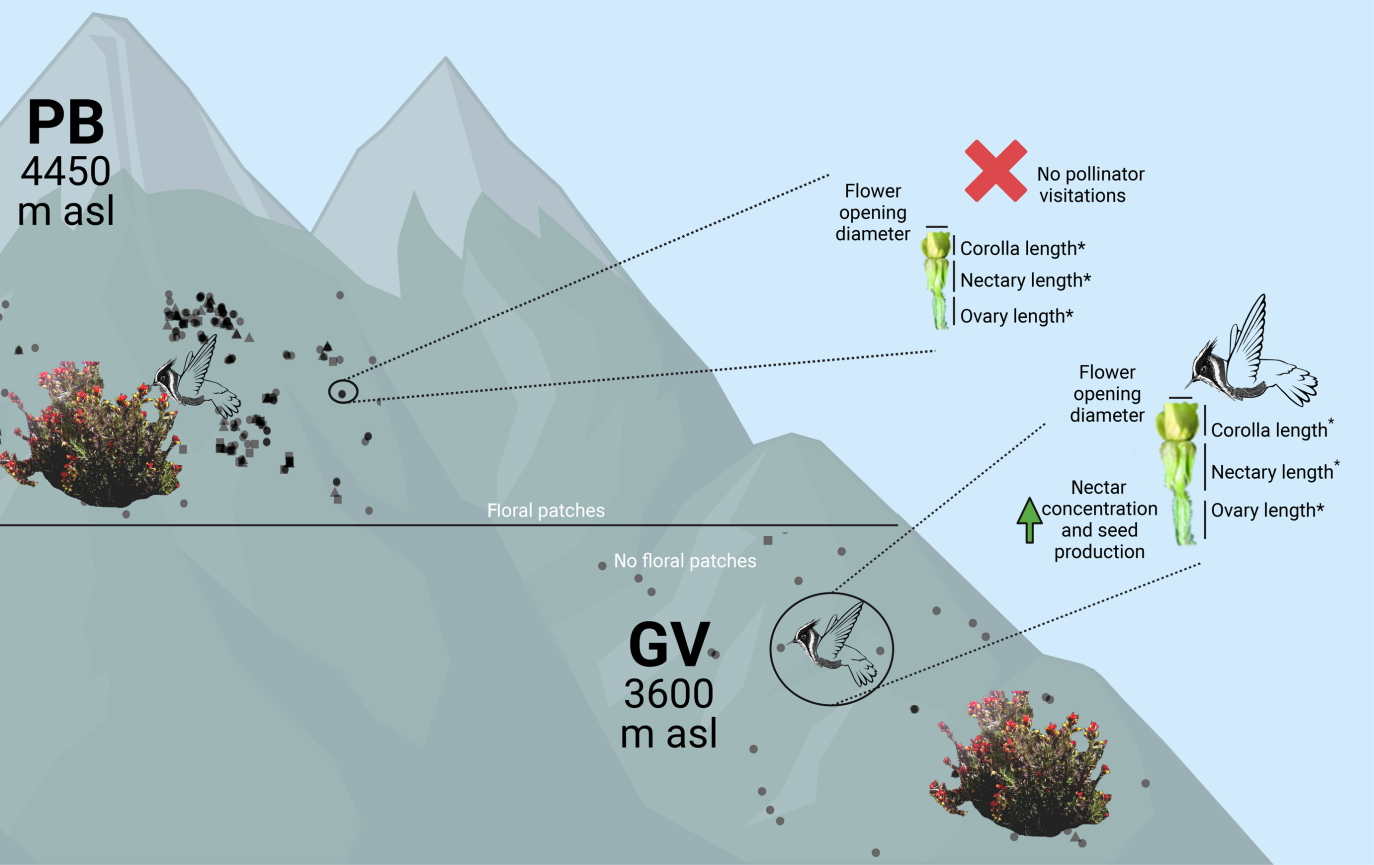
*Oenothera epilobiifolia* pollination ecology in PB and GV populations. Populations differ in altitude, mountain range and environmental conditions. The PB ecoregion is higher, colder and drier. Shape points represent the flower stage and spatial position on the ground: circle, triangle and square for the green, changing and red stages, respectively. In both communities, the other plant shown (*Castilleja* sp.) is present. In the GV population flowers are bigger, nectar concentration is higher, there are associated traits, pollinator visitations and higher seed production. On the contrary, in the PB population density and floral aggregation are higher, the traits associations do not exist and pollinators, even when present, did not visit *O. epilobiifolia* flowers, but did visit *Castilleja*'s flowers. Created with BioRender.com. (*) Trait significantly different between PB and GV.

## 4 DISCUSSION

This manuscript aimed to document the features and relationships between plant floral traits, plant mating system, pollinator composition and visitation and plant reproductive success across two plant populations that vary in their altitude. Floral traits are known to influence the composition and visitation rate of pollinators (Faegri & van der Pijil, 1979; Fenster et al., 2004; Sánchez‐Guillén et al., 2015; Varela et al., 2010) and thus we expected that change in floral traits across populations would have consequences for assembly of pollinators. Instead, we found that changes in floral traits do not change the assembly of pollinators, but the pollination ecology. We found that in both populations, the plant had different flower stages rather than distinct flower types, however, the duration of each stage varied between populations. Flowers in the lower population (GV) were larger and had higher sucrose concentration in their nectar. Pollinators were observed visiting flowers only in this lower population, primarily consisting of hummingbirds during the green flower stage. Flower density and aggregation were higher in the higher population and thus even though individual flowers have lower nectar concentration in this population, the overall energy content of nectar offered by the populations were similar. While both populations had similar capacities for self‐pollination in the absence of pollinators, an increased effect of pollinator presence on reproductive success was only observed in the lower population (GV), where we observed pollinator visits.

### 4.1 Role of other flower stages if only the green stage is relevant for pollination

Given that red corollas are more attractive for hummingbirds than green or yellow ones (Faegri & van der Pijil, 1979), we were surprised to find out that for *O. epilobiifolia* the fundamental events for pollination, nectar production and pollinator visitations, occurred only during the green stage. Therefore, pollinator visitations in *O. epilobiifolia* seem to be more influenced by the reward availability than by corolla colour. Red‐stage retention for at least 3 days is a phenomenon that has been reported in at least 200 angiosperm species and it is likely that even without rewards, these red flowers have an attractive effect on pollinators, especially considering the patchy aggregation of these flowers (Weiss, 1991). However, in this species, it has been reported that the red floral transition is part of the senescent process (Wagner & Hoch, 2005). Consequently, differences in the aggregation in patches is a product of the overlap of different flowering periods and changes in flower longevity. Similarly, in *Fuchsia excorticate* (Onagraceae), a plant with a reproductive system that is very similar to *O. epilobiifolia*, retention of the red‐stage was shown to be a physiological constraint (Delph & Lively, 1989). Nevertheless, considering the higher aggregation in the PB population, it remains to be tested whether in this system, the retention of the red flower and the aggregation still play a role attracting pollinators. This could be done by adding and removing artificial red flowers in GV and PB and evaluating its effect on visitation rates.

Regarding only the green stage, differences between populations in pollinator visitations and flower traits related to visits suggest a change in the pollination ecology of this plant. Nectar sucrose concentration in GV is within the ranges reported for ornithophilic flowers, while in PB it is under the lower extreme (Baker, 1975; Chalcoff et al., 2005; Kim et al., 2011). Faegri and van der Pijil (1979) proposed that in high and cold regions flower aggregation could be a strategy that would allow pollinators to make several visits with minimal effort. Nonetheless, contrary to Orchidaceae (Rodriguez‐Robles et al., 1992) and Passifloraceae (Pelayo et al., 2011) in which bonanza‐blank patterns improve the frequency of pollinator visits, the similarity between these localities in the energy nectar population offer indicates that the absence of pollinator visitations in PB is due to a negative effect of the bonanza‐blank pattern in nectar availability. We propose that this negative effect is produced when the bonanza‐blank is too high, that is when there are more ‘blanks’ than ‘bonanza’ flowers along with nectar of low quality per flower (such as in PB). If this is the case, the work involved in foraging (the search for a flower, the extraction and the movement to other flowers) is higher than the energy per visit reward. Consequently, from a population view, these flowers do not seem to be a desirable resource for pollinators. A good way of testing this hypothesis could be to alter the blank‐bonanza ratios by adding artificial flowers with sucrose solution in patches of different sizes and determining its influence on visitation rates.

### 4.2 Differences in pollination ecology driven by environmental heterogeneity

Our results showing smaller floral traits in the higher altitude population are in line with other research along altitudinal gradients (Monasterio, 1980; Sun et al., 2014). However, in contrast to others (Barrett et al., 2009; Glaettli & Barrett, 2008), we did not find evidence for more autogamous reproduction at higher elevations.

Traits of animal‐pollinated plant populations should respond in evolutionary time to intrinsic variation in pollinators and pollination success (Newman et al., 2014; van der Kooi et al., 2019). Because flower traits such as flower opening, nectary and corolla shapes, have been shown to affect pollinators' preferences (Brown et al., 2011; Fenster et al., 2004; Glaettli & Barrett, 2008; Sánchez‐Guillén et al., 2015; Varela et al., 2010), pollinators are known to exert selective pressures on those flower and nectar traits (Ornelas et al., 2007; Parachnowitsch et al., 2019; Staton & Young, 1994), resulting in mutualisms and trait‐matching (Leimberger et al., 2022). Thus, at any given time in a population, different phenotypes can be found with a group of traits states, which implies that selective pressures on them would occur simultaneously and, consequently, their responses would be concerted (Boberg & Ågren, 2009; Fenster et al., 2004). Reciprocal translocation experiments have been suggested as a way to determine the pressure that modulates the association of traits. If in the absence of the originally tested pressure the association is broken, then the pressure is responsible for that trait association (Staton & Young, 1994; Sun et al., 2014). In our case, the GV population is visited by pollinators and has associations between nectar volume and corolla, nectary length and flower opening. Pollinators could be having an effect on the frequency of the associated traits. By contrast, no pollinator visits nor trait associations were observed in the PB population.

Therefore, we suspect that populations in these two localities are tentative pollination ecotypes. On the one hand, in GV, although flowers are self‐compatible, pollinators promote cross‐fertilisation and phenotypes having pollinator‐related trait states are predominant in the population. On the other hand, in PB, the reproductive system is self‐fertilisation and the selection on trait states related to pollinator visitations must be low (Figure 6). An alternative explanation for this difference in pollinators‐mediated pollination is pollinators availability. Unfortunately, we had not directly quantified pollinators abundance in both ecoregions, but using the available reports in combination with our pollination visitation records we have confidence on this variation in seed production not resulting from differences in pollinators assembly *itself* as the Grant‐Stebbins model establishes for pollinations ecotypes (Grant, 1949; Johnson, 2010; Stebbins, 1970), but rather by drastic changes in environmental conditions.

We think this is the case because in PB the same pollinator assembly as in GV is present in the community. In Table 2 we show a modified list of an exhaustive description of the birds of the *páramos* of Mérida in three river basins in an altitudinal range between 2000 and 4200 m asl and June to September 2009 by Pelayo & Soriano, 2010. Chama river is nearby GV population and Motatán river nearby PB populations. This work was not only supported by the researchers direct observations but also by museum collections and ornithology books from the area. According to this list one of the two hummingbirds, *Oxypogon lindenii* (in the list as *Oxypogon guerinii*, name changed in 2013) (Collar & Salaman, 2013), that we found to be actively pollinating *O. epilobiifolia* in GV is also expected to occur in PB. Indeed, we have evidence of *O. lindenii* active in PB (4450 m asl), but visiting instead other flowers, no visiting *O. epilobiifolia* (Videos 2, 6 and 7). The other pollinator of *O. epilobiifolia* in GV reported in this work, *Metallura tyrianthina*, is not expected to be found in the higher population according to Pelayo & Soriano list (higher reported altitude 2800 m asl). We did not record *M. tyrianthina* in PB either. However, in the Andes, due to climate change, birds are expected to change their altitudinal range to higher altitudes due to increasing temperatures. Indeed, we have recorded *Colibri coruscans* in PB at 4450 m asl (Video 4), although the last reported higher altitude is 3600 m asl, 10 years ago. Therefore, we cannot reject the possibility of *M. tyrianthina* to be present in PB by the moment of this work. In addition, even if *M. tyrianthina* was not present in PB, our seed production experiment shows that in PB the presence of *O. lindenii* (which we have evidence of) does not influence seed production as it does in GV.

**TABLE 2:** Modified list of birds reported in Pelayo & Soriano, 2010 for three river basins that occur in the same elevantion range as our two population, GV and PB.

Specie	Reported elevation range (m asl)	River basin	GV (3600 m asl)	PB (4450 m asl)
*Phalacrocorax brasilianus*	3600	SD	1	
*Anas andium*	3200–3800	MO, SD	1	
*Anas discors*	3600	CH, SD	1	
*Anas cyanoptera septentrionalium*	4000	CH, SD		
*Aythya affinis*	400–3600	CH, SD	1	
*Bubulcus ibis*	300–3600	SD	1	
*Cochlearius cochlearius*	300–3600	SD	1	
*Phimosus infuscatus*	500–3600	SD	1	
*Ajaia ajaja*	300–3600	SD	1	
*Vultur gryphus*	2000–5000	CH, SD	1	1
*Cathartes aura*	3600	CH, SD	1	
*Geranoaetus melanoleucus australis*	3300–4500	SD	1	1
*Falco peregrinus*	3600	CH, SD	1	
*Heliornis fulica*	400–3600	SD	1	
*Pluvialis dominica*	1200–3600	CH, SD	1	
*Tringa melanoleuca*	4100	CH, SD		
*Tringa solitaria*	3600	CH, SD	1	
*Gallinago gallinago*	3600	CH, SD	1	
*Glaucidium jardinii*	2000–4000	MO, SD	1	1
*Chaetura pelágica*	350–3600	CH, SD	1	
***Colibri coruscans***	**600–3600**	**CH, MO, SD**	**1**	
***Eriocnemis vestitus***	**2700–3600**	**CH, MO**	**1**	
***Metallura tyrianthina oreopola***	**1700–3800**	**CH, MO, SD**	**1**	
***Oxypogon guerinii***	**3600–4500**	**CH, MO, SD**	**1**	**1**
*Piculus rivolii meridae*	1800–3700	MO, SD	1	
*Cinclodes fuscus*	3250–5000	CH, MO, SD	1	1
*Leptasthenura andicola*	3400–4400	CH, MO, SD	1	1
*Schizoeaca coryi*	2800–4100	CH, MO, SD	1	
*Asthenes wyatti mucuchiensi*	3600–4100	CH, MO, SD	1	
*Mercocerculus leucophrys gularis*	1350–3700	CH, MO, SD	1	
*Ochthoeca fumicolor*	2200–4200	CH, MO, SD	1	
*Myiotheretes fumigatus lugubris*	2200–3600	MO	1	
*Progne subis*	3600	CH, SD	1	
*Riparia riparia*	3600	CH, SD	1	
*Hirundo rustica*	3600	CH, MO, SD	1	
*Petrochelidon pyrrhonota*	3600	CH, SD	1	
*Cistothorus meridae*	3000–4100	CH, MO, SD	1	
*Turdus fuscater gigas*	1600–4200	CH, MO, SD	1	
*Anthus bogotensis*	2200–4100	CH, MO, SD		
*Dendroica palmarum*	3600	CH, SD	1	
*Dendroica castanea*	3600	CH, SD	1	
*Seiurus motacilla*	4200	CH, SD		
*Hemispingus superciliaris*	1900–3600	MO	1	
***Diglossa gloriosa***	**2500–4150**	**CH, MO, SD**	**1**	
*Catamenia inornata*	3250–4200	MO, SD	1	
*Phrygilus unicolor*	3000–4500	CH, MO, SD	1	1
*Atlapetes schistaceus*	2000–3800	CH, MO, SD	1	
*Zonotrichia capensis*	800–4000	CH, MO, SD	1	
*Carduelis spinescens*	2700–4100	CH, MO, SD	1	

*Note*: Possible and known pollinators are shown in bold. Highlighted in yellow, species that possible occur in the PB ecoregion. Highlighted in blue, species reported in this work.

Abbreviations: CH, Chama river; MO, Motatán river; SD, Santo Domingo river.

In a nutshell, we speculate that in these two populations the change in pollination strategy is not responding to the lack of pollinators or to the availability of different pollinator assembly, but to environmental change. In the higher ecoregion, PB, environmental conditions are considerably more extreme than in GV (Ataroff & Sarmiento, 2004; Monasterio, 1980; Petanidou et al., 1996). In these conditions, sucrose concentration in nectar must be answering to other pressures, e.g., water stress, high radiation, low temperature, poor soil, etc., which have been reported as influencing nectar quality (Cosacov et al., 2012; Parachnowitsch et al., 2019; Petanidou et al., 1996). Additionally, transitions to self‐fertilisation in plants have been detected at intraspecific levels, mainly with heterostyly flowers (Barrett, 2010). Further, Onagraceae has been a model for plant evolution (Krakos, 2011; Raven, 1979) because of its particular diversity in reproductive systems. Indeed, only in subclade B of *Oenothera* phylogeny, there are 13 transitions from self‐incompatibility to self‐compatibility (Krakos, 2011). Previous studies have shown that transitions to self‐compatibility of *Espeletias* ssp. have occurred because of an altitudinal gradient in the Venezuelan *páramos* (Berry & Calvo, 1989). As we find similar levels of autogamous self‐pollination at our high and lower elevation sites, pollinator visitation can only improve reproduction.

As we have found for these two populations and others in other systems (Barrett et al., 2009; Correa‐Lima et al., 2019; Gervasi & Schiestl, 2017; Paiaro et al., 2012), environmental conditions, beyond pressures by pollinators, can modulate changes in plant reproductive systems. Again, this hypothesis can be tested with transplant experiments and its effects on seed production.

### 4.3 High Tropical Mountain communities are highly dynamic, especially under climate change predictions

It is important to note that even in PB the pollinator dynamic could be different at another phenological moment when the energy reward offered by other plants is lower (Weinstein & Graham, 2017). Our results correspond to a period of *O. epilobiifolia* phenology, from August to October. Then, we suggest considering the whole phenology to better understand the plant‐pollinators relationship in a dynamic community. This is particularly relevant considering the high rate at which these systems are changing due to climate change (Correa‐Lima et al., 2019). As the temperature increases new communities are slowly being assembled towards higher altitudes (Llambí et al., 2021), as we have also found for the distribution range of *O epilobiifolia* and *C. coruscans*. Our results show that *O. epilobiifolia* and its pollinators relationship in these two eco‐regions is fragile and indeed has a profound effect on seed production. It is unknown what is the effect of the broken relationship on the side of the pollinators, as well as the change of the known pollinators altitudinal distribution. It is also unknown if other plants in these two eco‐regions community are responding in the same way to environmental heterogeneity. Also, if other populations of *O. epilobiifolia* are responding in the same way as we have found here.

In conclusion, the decrease of sucrose concentration in nectar, the reduction of flower parts with the lost of their trait associations, the increase of intensity and scale of flower aggregation in the PB population together with the absence of pollinator visitations, indicate a change in pollination ecology of *O. epilobiifolia* between the PB and GV populations. In GV, even with self‐compatibility, cross‐fertilisation mediated by pollinators selects for a phenotype that improves plant‐pollinator interaction resulting in higher seed production. Furthermore, in these two populations environmental heterogeneity modulates changes in plant‐pollinator relationships, rather than only pressures by pollinators. This hypothesis can be further tested with transplant experiments and by including pollinators distributions data, as well as population replicates at different altitudes.

## AUTHOR CONTRIBUTIONS

**Gisela T. Rodríguez‐Sánchez:** Conceptualization (supporting); data curation (lead); formal analysis (equal); investigation (equal); methodology (supporting); resources (supporting); visualization (lead); writing – original draft (lead); writing – review and editing (equal). **Roxibell C. Pelayo:** Conceptualization (lead); formal analysis (equal); investigation (equal); methodology (lead); resources (lead); supervision (lead); visualization (supporting); writing – review and editing (equal). **Pascual J. Soriano:** Conceptualization (supporting); methodology (supporting); resources (supporting); supervision (supporting); writing – review and editing (equal). **Tiffany M. Knight:** Supervision (supporting); writing – review and editing (equal).

## CONFLICT OF INTEREST STATEMENT

We declare that there are no conflicts of interest.

## Supporting information


Figure S1.

Figure S2.

File S1.

File S2.

## Data Availability

The datasets generated during and analysed during the current study, as well as the pipeline code in R, are available as supplementary material.
