# Supplementary material for: Intraspecific variation in pollination ecology due to altitudinal environmental heterogeneity
Source: Ecol Evol. 2024 Jun 18;14(6):e11553. doi: 10.1002/ece3.11553 (PMC11183924; doi:10.1002/ece3.11553)
Supplement: Supplementary file 1 — Figure S1. [file ECE3-14-e11553-s002.zip › ece311553-sup-0001-FigureS1.docx]

Figure S1. Flower density and aggregation. The six maps of 5 × 5 m plots in PB population. Points represent flowers and shape flower stage: circle, triangle and square for green, changing and red stages, respectively.
