# Supplementary figures and images for: Intraspecific variation in pollination ecology due to altitudinal environmental heterogeneity
Source: Ecol Evol. 2024 Jun 18;14(6):e11553. doi: 10.1002/ece3.11553 (PMC11183924; doi:10.1002/ece3.11553)

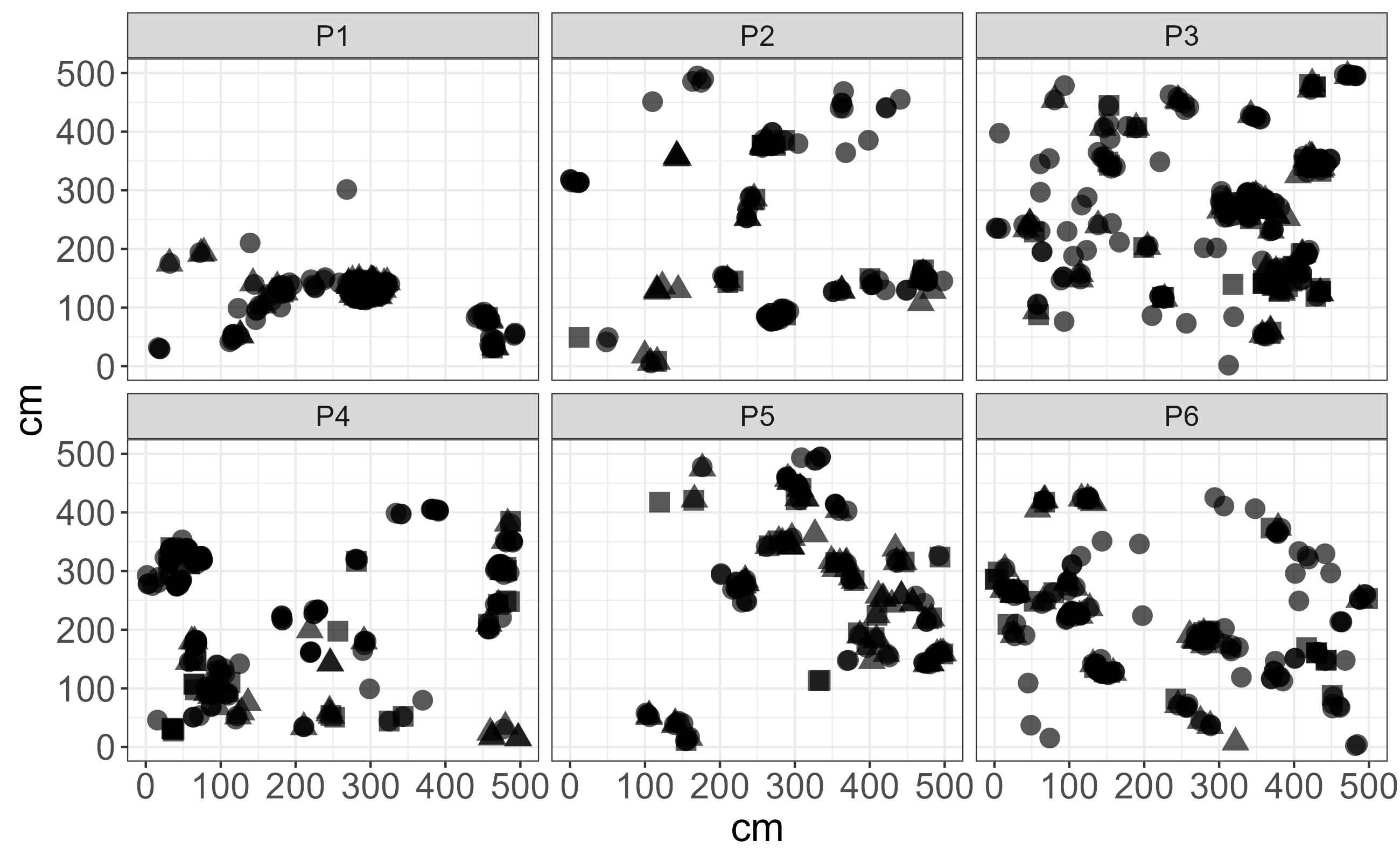

Supplement: Supplementary file 1 — Figure S1. [file ECE3-14-e11553-s002.zip › ece311553-sup-0001-FigureS1.tiff]

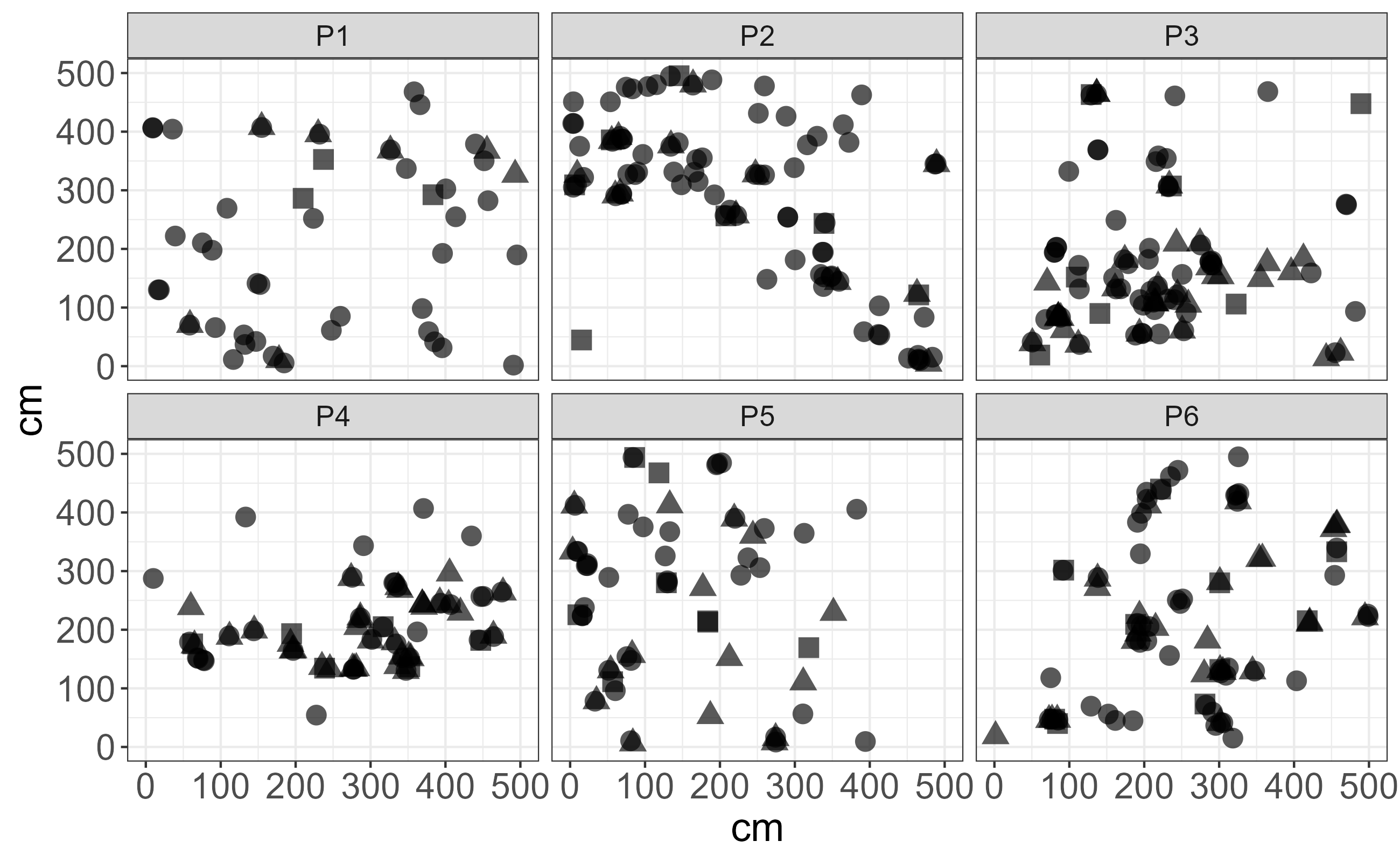

Supplement: Supplementary file 2 — Figure S2. [file ECE3-14-e11553-s001.zip › ece311553-sup-0002-FigureS2.tiff]
