# Supplementary material for: Intraspecific variation in pollination ecology due to altitudinal environmental heterogeneity
Source: Ecol Evol. 2024 Jun 18;14(6):e11553. doi: 10.1002/ece3.11553 (PMC11183924; doi:10.1002/ece3.11553)
Supplement: Supplementary file 3 — File S1. [file ECE3-14-e11553-s003.zip › ece311553-sup-0003-FileS1.docx]

File S1. Raw, generated and meta data.
