# Supplementary material for: Intraspecific variation in pollination ecology due to altitudinal environmental heterogeneity
Source: Ecol Evol. 2024 Jun 18;14(6):e11553. doi: 10.1002/ece3.11553 (PMC11183924; doi:10.1002/ece3.11553)
Supplement: Supplementary file 4 — File S2. [file ECE3-14-e11553-s004.zip › ece311553-sup-0004-FileS2.docx]

File S2. Pipeline processing code in R.
